# Anti-Inflammatory Effect of Auranofin on Palmitic Acid and LPS-Induced Inflammatory Response by Modulating TLR4 and NOX4-Mediated NF-κB Signaling Pathway in RAW264.7 Macrophages

**DOI:** 10.3390/ijms22115920

**Published:** 2021-05-31

**Authors:** Hyun Hwangbo, Seon Yeong Ji, Min Yeong Kim, So Young Kim, Hyesook Lee, Gi-Young Kim, Suhkmann Kim, JaeHun Cheong, Yung Hyun Choi

**Affiliations:** 1 Korea Nanobiotechnology Center, Pusan National University, Busan 46241, Korea; hbhyun2003@naver.com; 2 Department of Biochemistry, Dong-eui University College of Korean Medicine, Busan 47227, Korea; 14602@deu.ac.kr (S.Y.J.); ilytoo365@deu.ac.kr (M.Y.K.); 14731@deu.ac.kr (S.Y.K.); 14769@deu.ac.kr (H.L.); 3 Anti-Aging Research Center, Dong-eui University, Busan 47340, Korea; 4 Department of Marine Life Science, Jeju National University, Jeju 63243, Korea; immunkim@jejunu.ac.kr; 5 Center for Proteome Biophysics and Chemistry Institute for Functional Materials, Department of Chemistry, Pusan National University, Busan 46241, Korea; suhkmann@pusan.ac.kr; 6 Department of Molecular Biology, Pusan National University, Busan 46241, Korea

**Keywords:** auranofin, inflammation, macrophages, NF-κB/TLR4 signaling pathway, NOX4

## Abstract

Chronic inflammation, which is promoted by the production and secretion of inflammatory mediators and cytokines in activated macrophages, is responsible for the development of many diseases. Auranofin is a Food and Drug Administration-approved gold-based compound for the treatment of rheumatoid arthritis, and evidence suggests that auranofin could be a potential therapeutic agent for inflammation. In this study, to demonstrate the inhibitory effect of auranofin on chronic inflammation, a saturated fatty acid, palmitic acid (PA), and a low concentration of lipopolysaccharide (LPS) were used to activate RAW264.7 macrophages. The results show that PA amplified LPS signals to produce nitric oxide (NO) and various cytokines. However, auranofin significantly inhibited the levels of NO, monocyte chemoattractant protein-1, and pro-inflammatory cytokines, such as interleukin (IL)-1β, tumor necrosis factor-α, and IL-6, which had been increased by co-treatment with PA and LPS. Moreover, the expression of inducible NO synthase, IL-1β, and IL-6 mRNA and protein levels increased by PA and LPS were reduced by auranofin. In particular, the upregulation of NADPH oxidase (NOX) 4 and the translocation of the nuclear factor kappa-light-chain-enhancer of activated B cells (NF-κB) induced by PA and LPS were suppressed by auranofin. The binding between the toll-like receptor (TLR) 4 and auranofin was also predicted, and the release of NO and cytokines was reduced more by simultaneous treatment with auranofin and TLR4 inhibitor than by auranofin alone. In conclusion, all these findings suggested that auranofin had anti-inflammatory effects in PA and LPS-induced macrophages by interacting with TLR4 and downregulating the NOX4-mediated NF-κB signaling pathway.

## 1. Introduction

Inflammation is a biological response for protecting against and repairing damage from infections, injuries, and toxins [1,2]. Acute and chronic inflammation leading to excessive inflammation has been reported as a major cause of disease including non-alcoholic fatty liver disease, diabetes, inflammatory bowel disease, rheumatoid arthritis, vascular disease, and various types of cancer [3,4]. Inflammatory reactions that could trigger these diseases are initiated by the infiltration of activated inflammatory cells into the damaged site [5], where they produce cytokines and chemokines, amplifying the inflammatory response [6]. Macrophages play important roles in the immune defense system by releasing pro-inflammatory cytokines, including interleukin (IL)-1β, IL-6, and tumor necrosis factor (TNF)-α, and pro-inflammatory mediators, including nitric oxide (NO) and prostaglandin E2 (PGE_2_) [7]. For example, macromolecular ligand lipopolysaccharide (LPS) stimulates macrophages to produce pro-inflammatory cytokines and mediators [8]. Palmitic acid (PA) is a saturated fatty acid highly present in the blood of obese people that can induce an inflammatory response [9,10]. Moreover, several studies have reported that PA augmented the response to LPS, and low concentrations of LPS and PA caused chronic inflammation through the toll-like receptor (TLR) 4 signaling pathway [11,12,13].

As pattern recognition receptors, the TLR family is composed of transmembrane proteins and recognizes pathogen-associated molecular patterns such as lipoproteins, peptidoglycan, flagellin, and LPS [14]. TLR4 is the most studied member of the TLR family and plays an important role in initiating the inflammatory response by stimulating inflammatory cells [15,16]. Soluble LPS is recognized by a cluster of differentiation (CD) 14 and transmitted to myeloid differentiation factor 2 (MD2), a coreceptor that binds to TLR4 [17]. LPS recognition TLR4 is responsible for initiating inflammation by signal transduction to the myeloid differentiation primary response gene 88 and the nuclear factor kappa-light-chain-enhancer of activated B cells (NF-κB), which regulate the expression of inflammatory genes [18]. NF-κB is a transcription factor that plays a pivotal role in the expression of many genes regulating the immune and inflammatory responses [19]. In the absence of stimulation, NF-κB is inhibited by binding to the nuclear factor of kappa light polypeptide gene enhancer in B-cells inhibitor (IκB) in the cytoplasm [20,21]. After TLR4 is activated, IκB bound to NF-κB is phosphorylated by IκB kinase and then degraded, and NF-κB can be translocated to the nucleus [22]. NF-κB transferred to the nucleus induces the transcription of pro-inflammatory cytokines, chemokines, and additional inflammatory mediators.

The nicotinamide adenine dinucleotide phosphate (NADPH) oxidase (NOX) family was originally discovered in phagocytes and is a major source of superoxide produced by electron transfer to oxygen from NADPH [23,24]. The NOX complex stimulates the immune response by generating superoxide and plays critical roles in the development and progression of cancer and inflammatory disease [25,26,27]. Several previous studies have shown that the activation of NOX4 is associated with LPS-induced pro-inflammatory responses and activation of inflammasomes in a variety of cell lines, including human embryonic kidney cells, aortic endothelial cells, and smooth muscle cells [28,29,30,31]. Moreover, NOX4 upregulation in LPS-induced inflammation was related to activation of the NF-κB signaling pathway [32,33,34].

Auranofin, a lipophilic gold complex, was approved by the Food and Drug Administration for the treatment of rheumatoid arthritis [35]. In addition, auranofin has been reported to have potential efficacy in treating cancer, neurodegenerative disorders, and HIV/AIDS, as well as parasitic and bacterial infections [36]. Moreover, auranofin exerts anti-inflammatory properties by inhibiting the expression of pro-inflammatory cytokines through inactivation of the NF-κB signaling pathway [37,38]. However, studies on the effect of auranofin on the correlation between NOX4 and NF-κB increased by PA and LPS are still insufficient. In this study, the inhibitory effects of auranofin on PA and LPS-induced RAW264.7 macrophages were examined.

## 2. Results

### 2.1. PA Amplifies LPS-Stimulated Release of NO and Inflammatory Cytokines in RAW264.7 Macrophages

**Figure 1:**
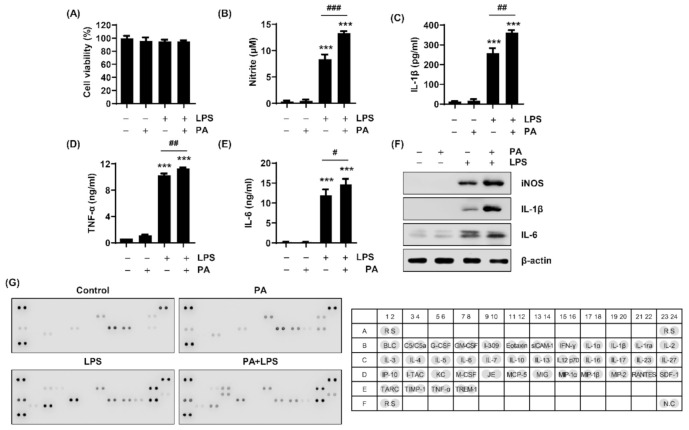
Inflammatory response induced in RAW264.7 macrophages by PA and LPS treatment. The cells were treated with 100 μM PA and 25 ng/mL LPS alone or co-treated for 24 h. (**A**) Cell viability was estimated by the 3-(4,5-Dimethylthiazol-2-yl)-2,5 diphenyltetrazolium bromide (MTT) assay. The absorbance was measured at 540 nm using a microplate reader. The levels of (**B**) NO, (**C**) IL-1β, (**D**) TNF-α, and (**E**) IL-6 in the cell culture media were assessed. The results are expressed as the mean ± standard deviation (SD) of three independent experiments. Statistical analysis was performed using one-way analysis of variance (ANOVA) with Tukey’s post-hoc test. *** *p* < 0.001 compared to untreated cells; ^#^ *p* < 0.05, ^##^ *p* < 0.01, and ^###^ *p* < 0.001 compared to LPS-treated cells. (**F**) The expressions of inducible NO synthase (iNOS), IL-1β, and IL-6 were measured by Western blot analysis. (**G**) Mouse cytokine array panel showing differences in inflammatory cytokines and chemokines in RAW264.7 macrophages. PA, palmitic acid; LPS, lipopolysaccharide.

To investigate the effect of auranofin on the inflammatory response of macrophages to PA and LPS treatment, the levels of NO and inflammatory cytokines were first investigated in RAW264.7 cells treated with PA and LPS alone or simultaneously. As shown in Figure 1, treatment with LPS alone significantly induced the release of NO and pro-inflammatory cytokines such as IL-1β, TNF-α, and IL-6 whereas treatment with PA alone did not. Additionally, the expression of inducible NO synthase (iNOS), IL-1β, and IL-6 were increased more with PA and LPS than each PA and LPS alone. Moreover, the results of the mouse cytokine array showed increased inflammatory cytokines and chemokines by PA and LPS. Interestingly, PA significantly enhanced the production of NO and inflammation-related cytokines induced by LPS. These results suggest that PA could further enhance the LPS-induced inflammatory response.

### 2.2. Auranofin Suppresses PA and LPS-Induced Expression Profiles of Pro-Inflammatory Genes

**Figure 2:**
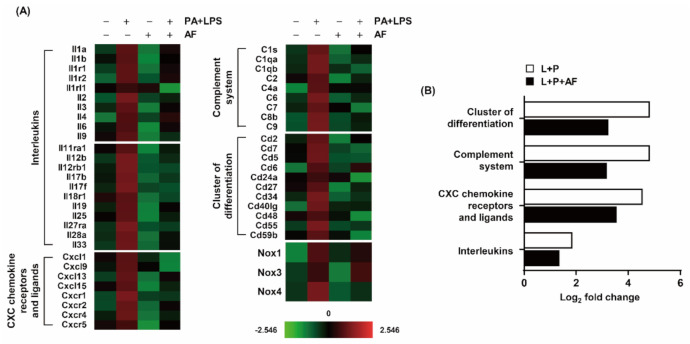
Upregulation of inflammatory genes in PA and LPS-induced RAW264.7 macrophages. Total RNA was extracted from the cells cultured under the conditions shown in figure inflammation-associated gene expression using the Nanostring nCounter Mouse Immunology Kit (NanoString Technologies, Inc., Seattle, WA, USA). (**A**) Heatmap representation of PA and LPS-treated RAW264.7 macrophages. Red represents upregulated expression levels and green represents down-regulated expression levels. (**B**) The bar graph shows log_2_ fold-changes by comparing the up-regulated expression of PA and LPS-treated cell and auranofin, PA, and LPS-treated cells. The data are the average of two independent experiments. AF, auranofin.

To confirm changes in genes related to the regulation of inflammation, RNA extracted from PA and LPS-treated RAW264.7 cells was assessed using NanoString nCounter analysis. As shown in the heatmap results in Figure 2, the expression of interleukins, CXC chemokine receptors (CXCR), CXC chemokine ligands (CXCL), complement system components, and the cluster of differentiation (CD) was increased in RAW264.7 cells by co-treatment with PA and LPS. PA and LPS enhanced the expression of interleukins and CXCR and CXCL groups by 1.86-log_2_-fold and 4.56-log_2_-fold, respectively, and the expression of complement factors and CD was elevated by 4.83-log_2_-fold and 4.82-log_2_-fold, respectively. However, auranofin reduced the expression of pro-inflammatory genes increased by PA and LPS (Figure 2B). Additionally, PA and LPS upregulated the expression of NOX1, 3, and 4 by 4.69-log_2_-fold, 5.42-log_2_-fold, and 4.24-log_2_-fold, respectively. Among them, the PA and LPS-induced expression of NOX4 was downregulated by auranofin. Specifically, the expression of NOX4 was decreased to 2.63-log_2_-fold. Therefore, these results demonstrated that the expression of pro-inflammatory genes was increased by PA and LPS, and auranofin inhibited their expressions. Furthermore, the involvement of NOX4 in modulating the inflammatory response could be suggested.

### 2.3. Auranofin Attenuates the Production of NO, Cytokine Secretion, and the Expression of Their Regulatory Genes

**Figure 3:**
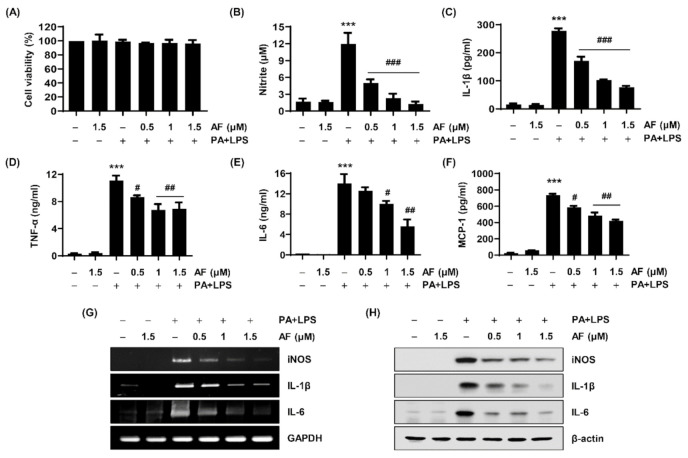
Effects of auranofin on the PA and LPS-induced inflammatory response in RAW264.7 macrophages. The cells were incubated with the indicated concentration of auranofin for 1 h and then treated with 100 μM PA and 25 ng/mL LPS for 24 h. (**A**) Cell viability was measured by the 3-(4, 5-Dimethylthiazol-2-yl)-2,5 diphenyltetrazolium bromide (MTT) assay. The secretion levels of (**B**) NO, (**C**) IL-1β, (**D**) TNF-α, (**E**) IL-6, and (**F**) MCP-1 were measured using Griess reagent and enzyme-linked immunosorbent assay (ELISA) kits (R&D Systems Inc., Minneapolis, MN, USA). The absorbance was measured using a microplate reader. The error bars represent the standard deviation of three independent experiments. Statistical analysis was performed using ANOVA with Tukey’s post-hoc test. *** *p* < 0.001 compared to untreated cells; ^#^ *p* < 0.05, ^##^ *p* < 0.01 and ^###^ *p* < 0.001 to PA and LPS-treated cells. The expression levels of iNOS, IL-1β, and IL-6 (**G**) proteins and (**H**) mRNA were measured by Western blot analysis and RT-PCR, respectively. β-actin and GAPDH were used as internal controls for RT-PCR and Western blot analyses, respectively.

Depending upon the gene expression analysis results, the levels of NO and pro-inflammatory cytokines secreted from RAW264.7 macrophages into the cell culture media were measured. As indicated in Figure 3B–F, PA and LPS significantly induced the release of NO as well as the secretion of cytokines such as IL-1β, TNF-α, IL-6, and monocyte chemoattractant protein-1 (MCP-1), but auranofin suppressed their production and secretion to a remarkable extent. Notably, PA (100 μM) and LPS (25 ng/mL), or PA and LPS with auranofin (0.5, 1, 1.5 μM) did not affect cell viability (Figure 3A). In a parallel experiment, the mRNA and protein expression levels of iNOS, IL-1β, and IL-6 were examined using a reverse transcription-polymerase chain reaction (RT-PCR) and Western blotting. As shown in Figure 3G,H, the expression of iNOS, IL-1β, and IL-6 was markedly increased by PA and LPS, and the expression was reduced by auranofin in a concentration-dependent manner.

### 2.4. Auranofin Decreases the PA and LPS-Induced Releases of Cytokines and Chemokines

**Figure 4:**
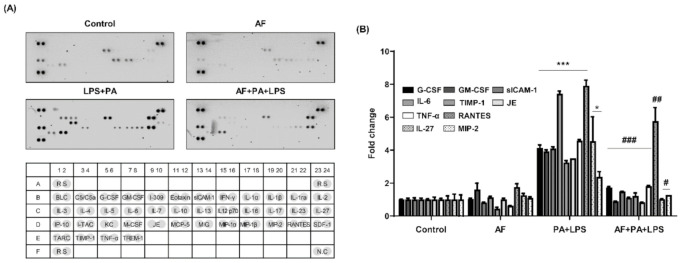
Effects of auranofin on cytokine production in PA and LPS-treated RAW264.7 macrophages. To measure cytokine secretion, the cell culture supernatants were collected and analyzed using the Proteome Profiler TM Mouse Cytokine Array kit. (**A**) Cytokine arrays and quantification of each spot. (**B**) The pixel densities were evaluated using ImageJ software (National Institutes of Health, Bethesda, MD, USA). The results represent the average of three replicates. The error bars represent the standard deviation of three independent experiments. Statistical analysis was performed using ANOVA with Tukey’s post-hoc test. * *p* < 0.05 and *** *p* < 0.001 compared to untreated cells; ^#^ *p* < 0.05, ^##^ *p* < 0.01 and ^###^ *p* < 0.001 to PA and LPS-treated cells. G-CSF, granulocyte colony-stimulating factor; GM-CSF, granulocyte-macrophage colony-stimulating factor; sICAM-1, soluble intercellular adhesion molecule-1; IL-6, interleukin-6; TIMP-1, metallopeptidase inhibitor-1; JE, known as monocyte chemoattractant protein-1(MCP-1); TNF-α, tumor necrosis factor-α; RANTES, regulated on activation, normal T cell expressed and secreted; IL-27, interleukin-27; MIP-2, macrophage inflammatory protein-2.

The levels of cytokines and chemokines were detected using a mouse cytokine array kit. Auranofin significantly decreased the levels of multiple cytokines in PA and LPS-treated RAW264.7 macrophages (Figure 4). Specifically, the high expression levels of G-CSF, CM-CSF, sICAM-1, IL-6, TIMP-1, JE, TNF-α, RANTES, IL-27, and MIP-2 in PA and LPS-treated RAW264.7 macrophages were reduced by auranofin. Consequently, these results indicate that auranofin exerted anti-inflammatory activity by inhibiting the secretion and expression of pro-inflammatory cytokines in PA and LPS-treated RAW264.7 macrophages.

### 2.5. PA and LPS Increases NOX4 Expression, and Auranofin Reduces the NOX4-Mediated Inflammatory Response

**Figure 5:**
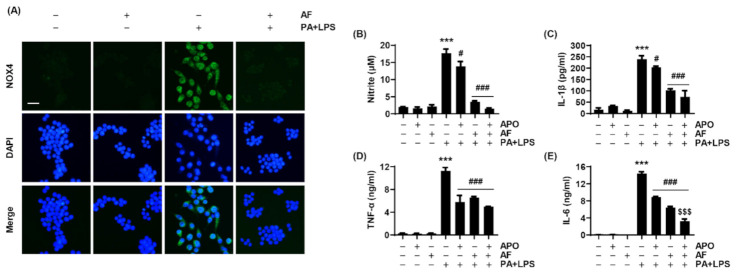
The association of NOX4 with the anti-inflammatory effects of auranofin. The cells were pre-treated with 100 μM apocynin and 1.5 μM auranofin for 1 h and with 100 μM PA and 25 ng/mL LPS for 1 h. (**A**) The expression of NOX4 (green fluorescence) was determined by immunofluorescence staining. Scale bar = 20 μm. The secretion levels of (**B**) NO, (**C**) IL-1β, (**D**) TNF-α, and (**E**) IL-6 were estimated using Griess reagent and ELISA kits. The error bars represent the standard deviation of three independent experiments. Statistical analysis was performed using ANOVA with Tukey’s post-hoc test (*n* = 3). *** *p* < 0.001 compared to untreated cells; ^#^ *p* < 0.05 and ^###^ *p* < 0.001 compared to PA and LPS-treated cells; ^$$$^ *p* < 0.001 compared to auranofin, PA, and LPS-treated cells.

The gene expression analysis showed that among NOX genes increased by PA and LPS, NOX4 was the most significantly decreased by auranofin. Hence, the expression of NOX4 protein was evaluated by immunofluorescent staining. Similar to the result of the gene expression analysis, Figure 5A shows that the expression of NOX4 increased by co-treatment with PA and LPS was markedly reduced by auranofin. Figure S1A shows that co-treatment with PA and LPS resulted in a higher expression of NOX4 than LPS or PA alone. Moreover, by examining the effect of apocynin, an NADPH oxidase inhibitor, on inhibiting the release of NO, IL-1β, TNF-α, and IL-6 by auranofin, we determined an association between the anti-inflammatory activity of auranofin and NOX4. As shown in Figure 5B–D, the production of NO, IL-1β, and TNF-α increased by PA and LPS tended to be reduced more by additional apocynin pre-treatment than by auranofin. Further, the secretion of IL-6 levels stimulated by PA and LPS was significantly decreased by apocynin treatment compared to auranofin (Figure 5E). In addition, as shown in Figure 2 and Figure 5, the expression of NOX4 was increased by PA and LPS, whereas auranofin reduced the expression of PA and LPS-induced NOX4 and decreased the secretion of pro-inflammatory cytokines.

### 2.6. Auranofin Inhibits the Translocation of NF-κB to the Nucleus by PA and LPS

**Figure 6:**
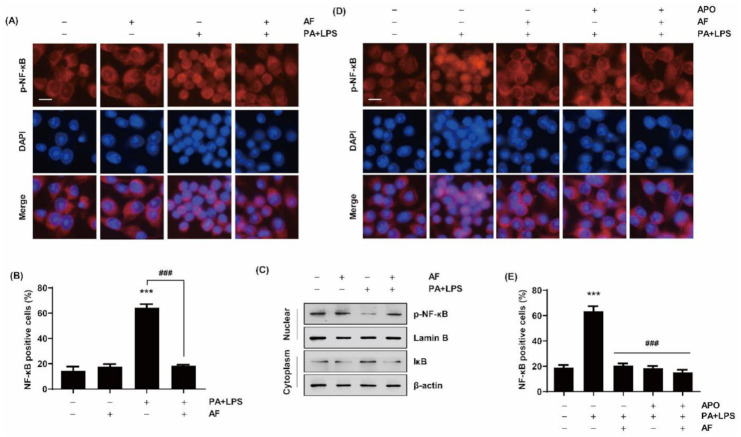
Effects of auranofin on the nuclear translocation of NF-κB in PA and LPS-treated RAW264.7 macrophages. (**A**) The cells were pre-treated with 1.5 μM auranofin for 1 h and with 100 μM PA and 25 ng/mL LP for 1 h. The cells were subjected to immunofluorescence staining with NF-κB antibody and representative fluorescence images are presented. Red fluorescence indicates the localization of NF-κB and blue fluorescence by 4′,6-diamidino-2-pheny (DAPI) staining allows visualization of the nuclei. (**B**) Graph showing the quantification of nuclear NF-κB-p65 positive staining in at least 300 counted cells presented as percentage ± SD. *** *p* < 0.001 compared to untreated cells; ### *p* < 0.001 compared to PA and LPS-treated cells. (**C**) After nuclear and cytoplasmic fraction, the expression level of NF-κB and IκB was assessed by Western blotting. Lamin B was used to normalize nuclear protein levels and β-actin was used to normalize cytoplasmic protein levels. (**D**) The cells were pre-treated with 100 μM apocynin and 1.5 μM auranofin for 1 h and with 100 μM PA and 25 ng/mL LPS for 1 h. The translocation of NF-κB was determined by immunofluorescence staining. (**E**) Graph showing the quantification of nuclear NF-κB-p65 positive staining in at least 300 counted cells presented as percentage ± SD. *** *p* < 0.001 compared to untreated cells; ^###^ *p* < 0.001 compared to PA and LPS-treated cells. Scale bar = 10 μm.

To confirm the association between the anti-inflammatory activity of auranofin and the NF-κB signaling pathway, the translocation of NF-κB to the nucleus was evaluated through immunofluorescence analysis. We verified that co-treatment with PA and LPS resulted in a higher expression of NF-κB in the nuclear than LPS or PA alone (Figure S1B). As shown in Figure 6A,B, p65 nuclear (red fluorescence) was translocated from the cytoplasm to the nucleus (blue fluorescence) by treatment with PA and LPS, but auranofin suppressed the translocation of NF-κB to the nucleus. Furthermore, we confirmed that auranofin markedly down-regulated the expression of p-NF-κB increased by treatment with PA and LPS (Figure 6C). Therefore, immunofluorescence data indicates that the anti-inflammatory properties of auranofin were mediated by reductions in the transcriptional activity of pro-inflammatory factors by inhibiting the activation of the NF-κB signaling system. Furthermore, we investigated the expression of NF-κB by apocynin treatment to confirm the association between NOX4 and NF-κB signaling and found that apocynin reduced the expression of NF-κB and inhibited translocation to the nucleus by PA and LPS (Figure 6D,E). These results suggest that NOX4 played an important role in the anti-inflammatory activity of auranofin in these cells and that NF-κB signaling was regulated by NOX4.

### 2.7. Auranofin Inhibits PA and LPS-Stimulated Inflammation by Interacting with the TLR4/MD2 Complex

**Figure 7:**
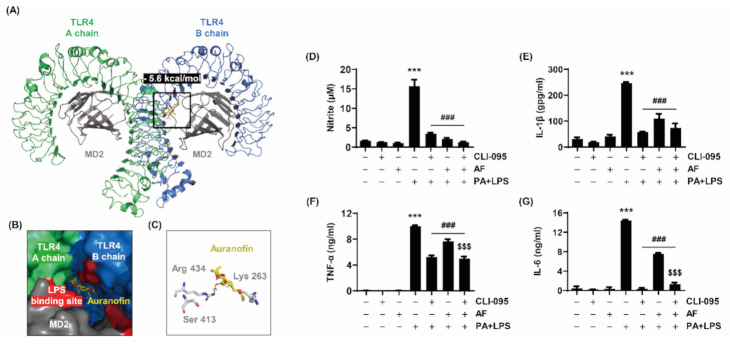
Molecular docking models of the TLR4/MD2 complex with auranofin. (**A**) Three-dimensional structure between the TLR4/MD2 complex and auranofin. TLR4 chain A, TLR4 chain B, MD2 and auranofin are indicated by green, blue, gray, and yellow sticks, respectively. The red-colored regions represent the LPS-binding residue. (**B**) Surface model representing auranofin bound to the surface pocket of the TLR4/MD2 complex. (**C**) The binding distance between auranofin and the Arg 434 residue of the TLR4/MD2 complex. (**D**–**G**) Relevance of TLR4 in the inhibitory effect of auranofin on inflammation due to PA and LPS co-treatment. The cells were pre-treated with 250 ng/mL CLI-095 and 1.5 μM auranofin for 1 h and with 100 μM PA and 25 ng/mL LPS for 24h. The levels of (**D**) NO, (E) IL-1β, (F) TNF-α, and (**G**) IL-6 released from the cells were measured using Griess reagent (Sigma-Aldrich Chemical Co., St. Louis, MO, USA) and ELISA kits. The error bars represent the standard deviation of three independent experiments. Statistical analysis was performed using ANOVA with Tukey’s post-hoc test. *** *p* < 0.001 compared to untreated cells; ^###^ *p* < 0.001 compared to PA and LPS-treated cells; ^$$$^ *p* < 0.001 compared to auranofin, PA, and LPS-treated cells.

To determine whether auranofin inhibition of RAW264.7 macrophages activated by PA and LPS involved the TLR4 signaling pathway, the interaction between auranofin and TLR4 was predicted, and changes in cytokine release were measured following treatment with a TLR4 inhibitor. First, LPS was removed from the structure of the TLR4/MD2/LPS complex obtained from the protein data bank (PDB) using PyMOL (Schrodinger, Inc., New York, NY, USA). Molecular modeling of the binding of the TLR4/MD2 complex, from which LPS was removed, to auranofin was analyzed using PyRx (The Scripps Research Institute, San Diego, CA, USA) to confirm whether auranofin competitively interacted with the LPS binding site of TLR4. As shown in Table 1, the binding of auranofin and TLR4 showed a high binding affinity of −5.6 kcal/mol. Auranofin was bound to Arg 434 of TLR4, which is correlated with LPS, and the distance between the bonds was 2.9 Å (Figure 7A–C). Furthermore, the release of NO and IL-1β tended to be reduced more by treatment with CLI-095 as an inhibitor of TLR4 than by auranofin (Figure 7D,E). Additionally, TNF-α and IL-6 were significantly reduced by CLI-095 treatment compared to auranofin (Figure 7F,G). Therefore, these results demonstrate that auranofin was associated with the TLR4 signaling pathway in the inhibition of the inflammatory response. In addition, the expression of NOX4 and activation of NF-κB were markedly up-regulated by co-treatment with LPS and PA, whereas its increment was greatly suppressed by CLI-905 (Figure S2A,B). This result indicates that it could be assumed that NOX4 and NF-kB could be regulated by TLR4. Furthermore, to verify whether the auranofin was involved in the intracellular pathway of TLR4-related signal, the expression and phosphorylation of MyD88 and p38 MAPK were investigated. Figure S2C shows that the expressions of MyD88 and p38 MAPK did not change under the same experimental conditions. Based on these results, we considered that auranofin has anti-inflammatory effects through the Myd88-indipendent TLR4/MD2 signaling pathway.

## 3. Discussion

Inflammation induced by the activation of macrophages acts as a defense system to protect against injury and harmful pathogens such as parasites, bacteria, and viruses [39,40]. These stimuli can activate macrophages, and activated macrophages produce and secrete many pro-inflammatory mediators, chemokines, and cytokines [7]. However, an excessive inflammatory response damages tissues and contributes to insulin resistance, obesity, metabolic syndrome, and the progression of disease related to chronic inflammation [16,41]. Therefore, inhibiting inflammation is an effective way to prevent the development and progression of various inflammation-related diseases. In this study, to induce an inflammatory response through macrophage activation, cells were treated with PA and LPS simultaneously. The results showed that LPS increased the release of pro-inflammatory mediator NO and cytokines such as IL-1β and TNF-α, and PA further increased these effects (Figure 1).

Auranofin is a gold-containing complex widely known as a treatment for rheumatoid arthritis. Recently, numerous studies have elucidated additional effects of auranofin, such as anti-inflammatory and anti-tumor effects [42,43]. The investigation most relevant to this study showed that the expression of IL-1β was induced in macrophages by LPS or bacteria, and the expression was inhibited by auranofin [44]. However, studies on the effect of auranofin on the inflammatory response caused by the LPS signals amplified by PA are insufficient. Therefore, this study is an attempt to demonstrate the mechanism by which auranofin inhibits the inflammatory response to PA and low-concentration LPS-stimulated macrophages.

A gene expression microarray was performed to investigate the influence of auranofin on PA and LPS-induced inflammatory factors such as interleukins, CXC chemokine receptors and ligands, the complement system, and cluster of differentiation. The results showed that auranofin reduced the PA and LPS-enhanced expression of pro-inflammatory chemokines and cytokines genes (Figure 2). This was also supported by the RT-PCR and Western blot results in which auranofin inhibited the expression of iNOS, the enzyme that produces NO, and the pro-inflammatory cytokines IL-1β and IL-6. Additionally, auranofin significantly reduced the PA and LPS-mediated release of NO, pro-inflammatory cytokines (IL-1β, TNF-α, IL-6, G-CSF, GM-CSF, and IL-27), and chemokines (MCP-1 and RANTES) from cells into the culture medium (Figure 3; Figure 4). These findings imply an association of NF-κB in the expression of proinflammatory-related genes. NF-κB is well known as a transcription factor responsible for inflammation by regulating the expression of pro-inflammatory cytokines and chemokine genes [19,45]. Yamashita et al., (2003) reported that auranofin could inhibit the production of NO and PGE2 by suppressing the translocation of NF-κB to the nucleus [46]. Recently, it has been reported that auranofin attenuated cardiac hypertrophy induced the apoptosis of multiple myeloma cells dependent upon the NF-κB signaling pathway [42,47]. The results of this study also show that auranofin diminished NF-κB activation in RAW264.7 macrophages stimulated with PA and LPS (Figure 6A). Therefore, the current results demonstrate that the increased nuclear translocation of NF-κB in PA and LPS-treated RAW264.7 macrophages resulted in the enhanced expression and release of pro-inflammatory mediators, cytokines, and chemokines, and that auranofin inhibited these effects. Furthermore, the NOX family proteins are key enzymes that cause the production of superoxide, and lately, studies have increasingly suggested an association with inflammation [48,49,50,51]. According to the gene expression microarray results in Figure 2, the expressions of NOX1, 3, and 4 were upregulated by treatment with PA and LPS. However, auranofin strongly reduced only the expression of NOX4 in untreated cells. NOX4 expression evaluated by immunofluorescence showed that auranofin decreased the expression of NOX4 increased by PA and LPS (Figure 5A). In addition, we used apocynin, a NOX inhibitor, to confirm the role of NOX4 in the PA and LPS-induced inflammatory response and the anti-inflammatory effects of auranofin and found that the PA and LPS-induced NO and proinflammatory cytokines were reduced by apocynin pre-treatment. This reduction showed a tendency to be much greater than that in cells treated with auranofin. Likewise, the translocation of NF-κB was also inhibited by both apocynin and auranofin (Figure 6B). Consequently, these results demonstrate the inhibitory effect of auranofin on PA and LPS-induced inflammation mediated by NOX4.

TLR4 is activated by recognizing LPS, resulting in an inflammatory response [14,16]. TLR4 complexed with MD2 and LPS is involved in initiating TLR4 signaling [15,17]. In this study, a molecular docking model was used to predict whether auranofin would competitively bind to the residues that LPS binds to TLR4. The LPS-binding residues in the TLR4/MD2 complex (PDB ID: 3VQ2) obtained from PDB were identified as Lys341, Lys360, Lys367, and Arg434 [52]. In addition, the binding affinity of the TLR4/MD2 complex and auranofin (compound identifier (CID) 70788951) was analyzed using PyRx (The Scripps Research Institute, San Diego, CA, USA). The analysis showed that auranofin and the Arg434 residue of the TLR4/MD2 complex were bound with a high affinity of −5.3 to −5.8 kcal/mol (Figure 7A–C). Nitrogen in the Arg434 of the TLR4/MD2 complex and the oxygen of auranofin were covalently bound at a distance of 2.9 Å. Therefore, these results indicate that auranofin inhibited the TLR4 signaling pathway by binding to the LPS binding site of TLR4. Although we have suggested a predictive model of TLR and auranofin, further studies are needed to verify that auranofin directly interacts with TLR4. Meanwhile, to confirm the role of TLR4 in the anti-inflammatory effect of auranofin, treatment with a TLR4 inhibitor, CLI-095, was compared with the anti-inflammatory effects of auranofin. The inflammatory response induced by PA and LPS was inhibited by CLI-095 and auranofin more than by auranofin treatment alone (Figure 7D–G). These findings show that PA and LPS induced pro-inflammatory gene expression and secretion of cytokines through the NOX4-mediated NF-κB signaling pathway, whereas auranofin suppressed PA and LPS-induced inflammatory responses.

## 4. Materials and Methods

### 4.1. Materials

Dulbecco’s modified Eagle’s medium (DMEM) and fetal bovine serum (FBS) were purchased from WELGENE (Gyeongsan, Korea). Six-well culture plates and four-well culture slides were purchased from SPL (Houston, TX, USA). Auranofin, LPS, PA, bovine serum albumin (BSA), DAPI, and CLI-095 were purchased from Sigma-Aldrich Chemical Co. (St. Louis, MO, USA). Dimethyl sulfoxide (DMSO) was purchased from Amresco, Inc. (Solon, OH, USA). 3-(4, 5-Dimethylthiazol-2-yl)-2,5 diphenyltetrazolium bromide (MTT) and TRIzol reagent were purchased from Invitrogen Life Technologies (Carlsbad, CA, USA). IL-1β, TNF-α, IL-6, and MCP-1 enzyme-linked immunosorbent assay (ELISA) kits, the Proteome Profiler TM Mouse Cytokine Array Kit, and apocynin were purchased from R&D Systems Inc. (Minneapolis, MN, USA). Skim milk and anti-iNOS were purchased from BD Biosciences (Franklin Lakes, NJ, USA). The One-Step RT-PCR PreMix Kit was purchased from iNtRON Biotechnology (Seongnam, Korea). Anti-IL-1β, IL-6, and NOX4 antibodies were purchased from Abcam (Cambridge, UK). Anti-β-actin antibody was purchased from Bioworld Technology, Inc. (Nanjing, China). Enhanced chemiluminescence (ECL) reagent was purchased from Thermo Fisher Scientific (Waltham, MA, USA). Anti-phosphorylated NF-κB was purchased from Cell Signaling Technology (Danvers, MA, USA). Secondary antibodies were purchased from Santa Cruz Biotechnology (Dallas, TX, USA).

### 4.2. Cell Culture

The RAW 264.7 cell line, derived from murine macrophages, was purchased from the Korea Cell Line Bank (Seoul, Korea). The cells were maintained in DMEM supplemented with 10% (*v/v*) FBS at 37 °C in a 5% CO_2_ humidified incubator at Core-Facility Center for Tissue Regeneration (Dong-eui University, Busan, Korea) as previously described [53]. 

### 4.3. Treatment of Chemicals

RAW 264.7 cells were seeded in six-well plates at 5 × 10^5^ cells per well and stabilized for 24 h. After that, the cells were treated with auranofin (0, 0.5, 1, and 1.5 μM) for 1 h and exposed to LPS (25 ng/mL) and PA (100 μM) for an additional 24 h. Auranofin was dissolved in DMSO to produce a 10 mM stock solution and stored at −20 °C until use. LPS was dissolved in distilled water at a concentration of 100 μg/mL and PA was dissolved in ethyl alcohol and BSA at a concentration of 100 mM and stored at 4 °C until use. Apocynin and CLI-095 were dissolved in DMSO, and these were treated 1 h before auranofin treatment.

### 4.4. MTT Assay

The effects of auranofin, LPS, and PA on the cell viability and proliferation were evaluated by an MTT assay [54]. After 24 h of treatment incubation, the medium in each well was removed by aspiration and replaced with 0.5 mg/mL of MTT solution. Then, the well plates were incubated at 37 °C for another 2 h. Subsequently, all supernatant was removed, the formazan crystals were dissolved in DMSO, and the absorbance was measured at a wavelength of 540 nm using a microplate reader (Molecular Devices, Sunnyvale, CA, USA). The results are expressed as percentages of the treated group compared to the control group.

### 4.5. Assessment of Nitrite Production

NO production was estimated by the quantity of nitrite released using colorimetric assays with Griess reagent. In brief, the cell culture supernatant was mixed with an equal volume of Griess reagent (0.5% sulfanilamide and 0.05% *N*-1-naphthylethylenediamine) and incubated in 96-well plate for 10 min at room temperature. The absorbance was measured at 540 nm using a microplate reader and calculated by comparison to a sodium nitrite (NaNO_2_) standard curve [55].

### 4.6. Measurement of Cytokine Level

To measure the cytokine levels, cell culture supernatants were collected and frozen at −80 °C until use. The levels of IL-1β and MCP-1 in the culture medium were measured, and those of TNF-α and IL-6 were measured by diluting the culture medium. The release of IL-1β, TNF-α, IL-6, and MCP-1 were quantified using mouse-specific ELISA kits according to the manufacturer’s instructions [56]. The absorbance was measured at a wavelength of 450 nm using a microplate reader.

### 4.7. Analysis of Gene Expression in the Immune Response

The analysis of gene expression in the immune response was performed using the NanoString nCounter™ Mouse Inflammation v2 Panel (NanoString Technologies, Inc., Seattle, WA, USA). For sample preparation, total RNA was extracted from RAW264.7 cells after treatment. The quantity and quality of the extracted RNA were measured using a DS-11 spectrophotometer (DeNovix Inc., Wilmington, DE, USA) and an AATI Fragment Analyzer (Agilent Technologies, Santa Clara, CA, USA). The samples with sufficient RNA purity, demonstrated by an RNA concentration of 20 ng/μL or more and an acceptable 260 nm/280 nm absorbance ratio were used. Total RNA was hybridized to a reporter-capture probe at 65 °C for 24 h. After the hybridization reaction, the samples were loaded onto an nCounter cartridge (NCT-120) and the data were gathered by the nCounter Prep Station and the nCounter Digital Analyzer. The raw data were normalized to the housekeeping gene expression and expressed as fold-change. A heatmap was generated using the normalized data.

### 4.8. RT-PCR Analysis

Total RNA was isolated using TRIzol reagent. cDNA was synthesized from the extracted RNA and iNOS, IL-1β, IL-6, and glyceraldehyde 3-phosphate dehydrogenase (GAPDH) genes were amplified using a One-Step RT-PCR PreMix Kit. The primers used for amplification are shown in Table 2. The PCR cycling conditions were 45 °C for 30 min, 94 °C for 5 min, denaturation at 94 °C for 30 s, annealing at 55 °C (or 65 °C) for 30 s and elongation at 72 °C for 30 s for 30 cycles. The amplification products were maintained at 4 °C until further use. The amplified DNA products were electrophoresed on 2% agarose gels and visualized using the Fusion Solo S system (Vilber Loumat, Collégien, France) after ethidium bromide staining as previously described [57].

### 4.9. Western Blot Analysis

To extract proteins, the cells were washed with cold phosphate-buffered saline (PBS) and lysed with lysis buffer, followed by centrifugation at 18,340× *g* for 30 min at 4 °C as previously described [58]. Equal amounts of protein were separated by sodium dodecyl sulfate-polyacrylamide gel electrophoresis (SDS-PAGE) and transferred to nitrocellulose membranes. Subsequently, the membranes were blocked with 5% skim milk in PBS containing 0.1% Tween 20 (PBST) for 1 h at room temperature, and incubated with primary antibodies overnight at 4 °C. Following three washes with PBST, the membranes were reacted with secondary antibodies (anti-mouse or anti-rabbit) for 1 h at room temperature, and the immunoreactive bands were visualized using ECL. The blots were detected and analyzed by a Fusion Solo S system.

### 4.10. Cytokine Array Analysis

The immune responses of RAW264.7 cells cultured under various conditions were analyzed using the Proteome Profiler^TM^ Mouse Cytokine Array Kit according to the manufacturer’s instructions. Briefly, the cytokine array membranes were blocked for 1 h, and incubated with the cell culture supernatant-antibody mixture overnight at 4 °C. Subsequently, the array membranes were washed with the wash buffer provided in the kit and incubated in streptavidin-horseradish peroxidase for 30 min. The dot blots were reacted with ECL and chemiluminescence was detected using Fusion Solo S system. Densitometric analysis of the data was performed using ImageJ^®^ software (v1.48, National Institutes of Health, Bethesda, MD, USA).

### 4.11. Immunofluorescence

RAW264.7 cells were seeded in 4-well cell culture slides and stabilized for 24 h. The cells were pre-treated with auranofin or apocynin, an NADPH oxidase inhibitor, for 1 h and then treated with or without LPS and PA for another 1 h. After treatment, the cells were fixed with ice-cold methanol for 10 min and washed with PBS. Subsequently, the cells were blocked using 5% BSA with PBS-T (PBS containing 0.1% Triton X) for 1 h and then incubated with anti-p-NF-κB (1:100 in 2.5% BSA in PBS-T) and anti-NOX4 (1:100 in 2.5% BSA in PBS-T) at 4 °C overnight. The cells were washed with PBS-T and incubated with a secondary antibody (goat anti-rabbit IgG cross-absorbed secondary antibody conjugated to Alexa Fluor 594 or Alexa Fluor 488) for 1 h. After washing with PBS, the cells were counterstained with DAPI for 20 min. Cell fluorescence was observed using an EVOS FL Auto 2 imaging system (Thermo Fisher Scientific). The quantitative analysis of mean number of cells was performed by the image J^®^ software.

### 4.12. Molecular Docking Prediction for TLR4 and Auranofin Interaction

To predict the interaction of auranofin and TLR4, the three-dimensional structure of the TLR4/MD2 complex was obtained from the PDB and that of auranofin was obtained from the National Center for Biotechnology Information PubChem compound database. The PDB ID code and PubChem CID are shown in Table 1. In the docking study, binding affinity was calculated by the PyRx virtual screening program (The Scripps Research Institute, San Diego, CA, USA, https://pyrx.sourceforge.io, accessed 14 July 2020) and the binding of TLR4 and auranofin was analyzed and visualized by the PyMOL molecular graphics system (Schrodinger, Inc., New York, NY, USA, https://pymol.org, accessed 14 July 2020).

### 4.13. Statistical Analysis

All statistical analysis was performed with GraphPad Prism 8.4.2 (GraphPad Software, Inc., San Diego, CA, USA) using one-way analysis of variance (ANOVA) for multiple comparisons, followed by Tukey’s post hoc test. All numerical data are presented as the mean ± standard deviation (SD) of at least triplicate experiments. Values of *p* < 0.05 were considered to be statistically significant.

## 5. Conclusions

**Figure 8:**
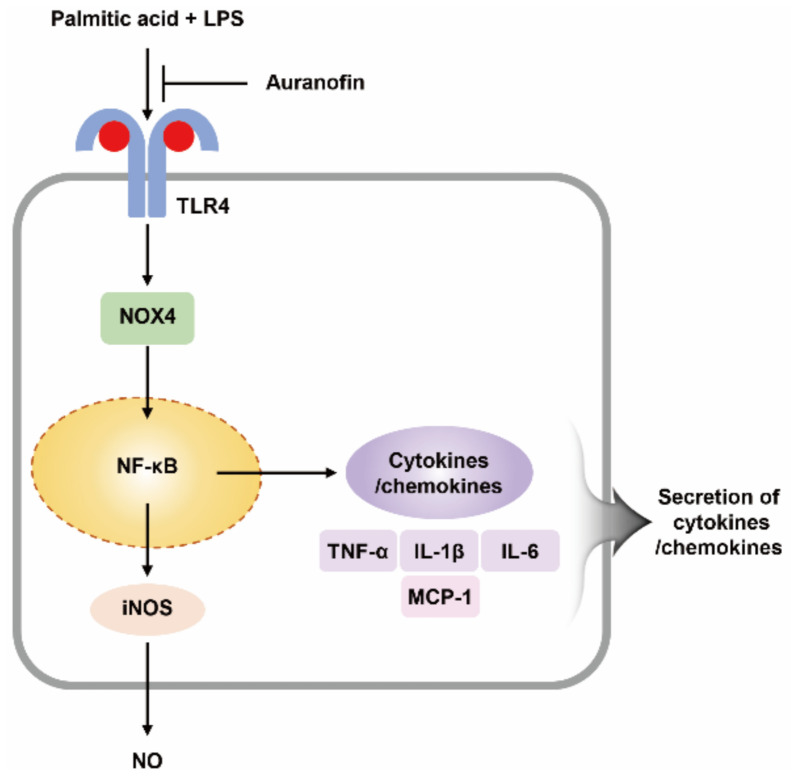
Graphical summary of the anti-inflammatory effects of auranofin on PA and LPS-stimulated RAW264.7 macrophages.

In conclusion, the study suggested the effects of auranofin on the inflammatory response induced by PA and low concentration of LPS in RAW264.7 macrophages. The results show that auranofin decreased the secretion and expression of proinflammatory cytokines such as IL-1β, TNF-α, and IL-6 induced by palmitic acid and LPS. Moreover, the translocation of NF-κB to the nucleus and the expression of NOX4 levels increased by palmitic acid and LPS were inhibited by auranofin. Furthermore, auranofin was predicted in its the interaction with TLR4, and the inflammatory response was reduced further by treatment with TLR4 inhibitor than by using auranofin alone. These results demonstrate that auranofin has anti-inflammatory properties in palmitic acid and LPS-induced macrophages through its interaction with TLR4 and downregulation of the NOX4-mediated NF-κB signaling pathway (Figure 8). Although further studies are needed to elucidate the mechanism by which TLR4 signaling modulates NOX4, the results of this study support the recent findings that auranofin has strong anti-inflammatory effects.

## Figures and Tables

**Table 1:** Binding data of auranofin and the TLR4/MD2 complex.

Molecule	Compound	Binding Affinity (kcal/mol)	Binding Site	Distance (Å)
Toll-like receptor 4 (PDB ID 3VQ2)	Auranofin (CID 70788951)	–5.6	*N* (Arg 434)	2.9

**Table 2:** List of RT-PCR primers.

Gene		Primers	Tm (°C)
iNOS	F	CGT GTT TAC CAT GAG GCT GA	55
	R	GCT TCA GGT TCC TGA TCC AA	
IL-1 β	F	AAT CTC GCA GCA GCA CAT CA	55
	R	AGC CCA TAC TTT AGG AAG AC	
IL-6	F	CTG GTG ACA ACC ACG GCC TTC CCT A	65
	R	ATG CTT AGG CAT AAAC GCA CTA CCT T	
GAPDH	F	GAA GAG TGG GAG TTG CTG TT	55
	R	GGA GAA ACC TGC CAA GTA TG	

F, forward; R, reverse; iNOS, inducible oxide synthase; IL-1β, interleukin-1β; IL-6, interleukin-6; GAPDH, glyceraldehyde 3-phosphate dehydrogenase; Tm, melting temperature.

## Data Availability

The data presented in this study are available within the article. Other data that support the findings of this study are available upon request from the corresponding authors.

## Supplementary Materials

The following are available online at https://www.mdpi.com/article/10.3390/ijms22115920/s1, Figure S1. The effect of LPS and/or PA treatment on the expression of NOX4 and activation of NF-κB in RAW264.7 cells, Figure S2. Modulation of NOX4 and NF-κB by TLR4 inhibitor (CLI-095).
