# Supplementary material for: Anti-Inflammatory Effect of Auranofin on Palmitic Acid and LPS-Induced Inflammatory Response by Modulating TLR4 and NOX4-Mediated NF-κB Signaling Pathway in RAW264.7 Macrophages
Source: Int J Mol Sci. 2021 May 31;22(11):5920. doi: 10.3390/ijms22115920 (PMC8198732; doi:10.3390/ijms22115920)
Supplement: Supplementary file 1 [file ijms-22-05920-s001.zip › ijms-1228366-supplementary.pdf]

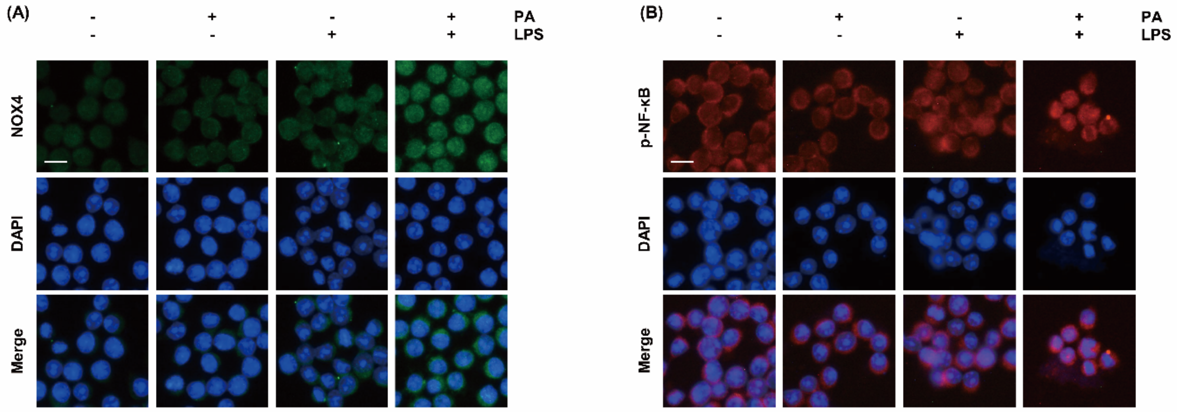

Figure S1. The effect of LPS and/or PA treatment on the expression of NOX4 and activation of NF-κB in RAW264.7 cells. The cells were treated with 100 μM PA and 25 ng/ml LPS alone or co-treated for 24 h. (A) The expression of NOX4 (green fluorescence) was determined by immunofluorescence staining. (B) The cells were subjected to immunofluorescence staining with NF-κB antibody and representative fluorescence images are presented. Red fluorescence indicates the localization of NF-κB. Blue fluorescence by DAPI staining allows visualization of the nuclei. Scale bar = 10 μm.

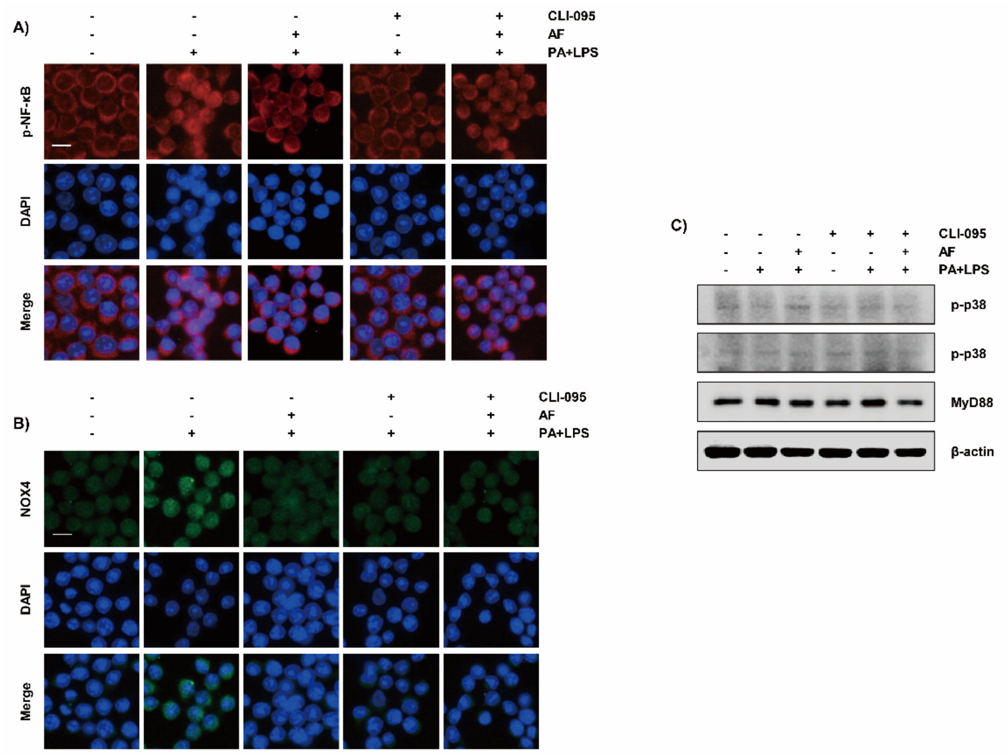

Figures S2. Modulation of NOX4 and NF-κB by TLR4 inhibitor (CLI-095). The cells were pre-treated with 100 μM apocynin and 1.5 μM auranofin for 1 h and with 100 μM PA and 25 ng/ml LPS for 1 h. (A) The expression of NOX4 (green fluorescence) was determined by immunofluorescence staining. (B) The cells were subjected to immunofluorescence staining with NF-κB antibody and representative fluorescence images are presented. Red fluorescence indicates the localization of NF-κB and blue fluorescence by 4',6-diamidino-2-pheny (DAPI) staining allows visualization of the nuclei. Scale bar = 10 μm. (C) The expression levels of phosphorylated p38 and MyD88 were estimated using Western blotting. β-actin was used as internal controls for Western blot analyses.
